# The pre-ECMO simplified acute physiology score II as a predictor for mortality in patients with initiation ECMO support at the emergency department for acute circulatory and/or respiratory failure: a retrospective study

**DOI:** 10.1186/s13049-015-0135-x

**Published:** 2015-08-17

**Authors:** Kun Il Kim, Hee Sung Lee, Hyoung Soo Kim, Sang Ook Ha, Won Yong Lee, Sang Jun Park, Sun Hee Lee, Tae Hun Lee, Jeong Yeol Seo, Hyun Hee Choi, Kyu Tae Park, Sang Jin Han, Kyung Soon Hong, Sung Mi Hwang, Jae Jun Lee

**Affiliations:** Department of Thoracic and Cardiovascular Surgery, Hallym University Sacred Heart Hospital, Hallym University Medical Center, 22, Gwanpyeong-ro 170 beon-gil, Donan-gu, Anyang-si, Gyeonggi-do 431-070 South Korea; Department of Emergency Medicine, Hallym University Medical Center, Kyoungki-do, South Korea; Department of Emergency Medicine, Hallym University, Chuncheon, South Korea; Division of Cardiology, Department of Internal Medicine, Hallym University, Chuncheon, South Korea; Department of Anesthesiology, School of Medicine, Hallym University, Chuncheon, South Korea; Department of Anesthesiology, School of Medicine, Hallym University, Chuncheon, South Korea; Department of Anesthesiology, School of Medicine, Hallym University, Chuncheon, South Korea

**Keywords:** SAPS II, Extracorporeal membrane oxygenation, Emergency department, Circulatory failure, Respiratory failure

## Abstract

**Background:**

In the emergency department (ED), extracorporeal membrane oxygenation (ECMO) can be used as a rescue treatment modality for patients with refractory circulatory and/or respiratory failure. Serious consideration must be given to the indication, and the PRESERVE and RESP scores for mortality have been investigated. However these scores were validated to predict survival in patients who received mainly veno-venous (VV) ECMO in the intensive care unit. The aim of the present study was to investigate the factors that predicted the outcomes for patients who received mixed mode (veno-arterial [VA] and VV) ECMO support in the ED.

**Methods:**

This single center retrospective study included 65 patients who received ECMO support at the ED for circulatory or respiratory failure between January 2009 and December 2013. Pre-ECMO SAPS II and other variables were evaluated and compared for predicting mortality.

**Results:**

Fifty-four percent of patients received ECMO-cardiopulmonary resuscitation (E-CPR), 31 % received VA and V-AV ECMO, and 15 % received VV ECMO. The 28-day and 60-month mortality rates were 52 % and 63 %. In the multivariate analysis, only the pre-ECMO Simplified Acute Physiology Score II (SAPS II) (odd ratio: 1.189, 95 % confidence interval: 1.032–1.370, *p* = 0.016) could predict the 28-day mortality. The area under the receiver operating characteristic curve and the optimal cutoff value for pre-ECMO SAPS II in predicting 28-day mortality was 0.852 (95 % CI: 0.753–0.951, *p* < 0.001) and 80 (sensitivity of 97.1 % and specificity of 71.0 %), respectively. Validation of the 80 cutoff value revealed a statistically significant difference for the 28-day and 60-month mortality rates in the overall, E-CPR, and VA groups (28-day: *p* < 0.001, *p* = 0.004, p = 0.005; 60-month: *p* < 0.001, *p* = 0.004, *p* = 0.020). In the Kaplan-Meier analysis, the 28-day and 60-month survival rates were lower among the patients with a pre-ECMO SAPS II of ≤80, compared to those with a score of >80 (both, *p* < 0.001).

**Conclusion:**

The pre-ECMO SAPS II could be helpful for identifying patients with refractory acute circulatory and/or respiratory failure who will respond to ECMO support in the ED.

## Background

Patients with acute circulatory and/or acute respiratory failure in the emergency department (ED) must be diagnosed rapidly and accurately, and an optimal treatment plan must be established according to their initial diagnosis. Unfortunately, many patients’ condition rapidly deteriorates before an accurate diagnosis, despite the use of a vasopressor and mechanical ventilator. In this context, extracorporeal membrane oxygenation (ECMO) can be used as a rescue treatment to stabilize their condition and provide additional time to reach an accurate diagnosis, which ultimately leads to improvements in survival rates.

ECMO has recently become increasingly popular for treating patients with acute heart and/or respiratory failure or arrest who do not respond to conventional treatment [1–9]. However, serious consideration must be given to the therapeutic indication for ECMO, due to the high economic cost and labor intensive nature. Therefore, several mortality risk score systems (PRESERVE and RESP) have been investigated for identifying patients who are indicated for ECMO support [10, 11]. However these scores were validated to predict survival in patients who received mainly veno-venous (VV) ECMO in the intensive care unit (ICU). The aim of the present study was to investigate the factors that predict the outcomes for patients who received mixed mode (veno-arterial [VA] and VV) ECMO support in the ED for circulatory or respiratory failure.

## Methods

### Patient enrollment criteria

**Fig. 1:**
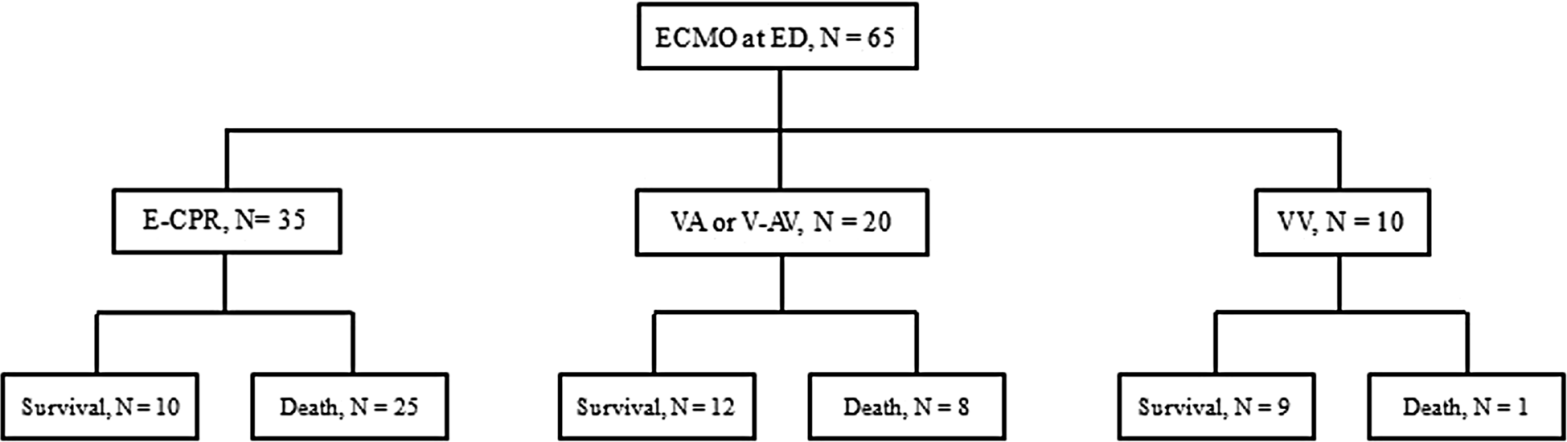
Flow chart of the study population and outcomes at 28 days. ED: emergency department; ECMO: extracorporeal membrane oxygenation, E-CPR: ECMO-cardiopulmonary resuscitation, VV: veno-venous, V-AV: veno-arteriovenous

This study was approved by the institutional review board of Hallym University Chuncheon Sacred Heart Hospital, and the requirement for informed consent was waived, due to the retrospective design. Our search identified 65 patients with acute circulatory and/or respiratory failure who did not respond to conventional treatment and received ECMO at the ED between January 2009 and December 2013 (Fig. 1). The indications for VA ECMO were (1) refractory cardiogenic, septic, or neurogenic shock, with a systolic blood pressure of <80 mmHg, despite appropriate conventional treatment; (2) cardiac arrest that did not present with return of spontaneous circulation within 10 min of cardiopulmonary resuscitation (CPR); and (3) recurrent cardiac arrest within 20 min of return of spontaneous circulation after CPR. The indications for VV ECMO were (1) a ratio of <100 for partial arterial oxygen pressure to fractional inspired oxygen concentration (PaO_2_/FiO_2_) on FiO_2_ 1.0 or (2) a pH of <7.20 that was caused by the lack of correction in CO_2_ retention, despite appropriate conventional treatment for acute respiratory failure.

In addition, patients who were predicted to require a high dose of inotropic agents (norepinephrine >0.25 μg/kg/min) during VV ECMO (due to acute respiratory failure) received V-AV ECMO. Furthermore, if a patient presented with upper limb hypoxia, due to a lack of lung or heart function recovery in the VA mode, they received V-AV or VV ECMO. Patients did not receive ECMO in cases of un-witnessed cardiac arrest, terminal malignancy, or if they were unlikely to regain normal function after general recovery.

### Patient data collection

Electronic medical records were reviewed. Pre-ECMO characteristics (age, sex, body mass index, medical history, diagnosis, arrest [location, CPR time and complication, ECMO-cardiopulmonary resuscitation], laboratory finding, SOFA, pre-ECMO SAPS II, and door-to-ECMO time) and duration and post-ECMO characteristics (ECMO modes, anticoagulation, duration, continuous renal replacement therapy, transfusion, length of stay) were retrospectively calculated.

### ECMO equipment

Three types of centrifugal pumps were used for ECMO. Until May 2010, the Capiox Emergency Bypass System® (Terumo, Inc., Tokyo, Japan) and Bio-pump® (Medtronic Inc., Minneapolis, USA) were used at our institution. However, since June 2010, the Centrifugal Rotaflow Pump® (Maquet Inc., Hirrlingen, Germany) has been used for most patients. The cannulae were 17–21-Fr arterial cannulae (DLP®, Biomedicus®; Medtronic Inc., or RMI®; Edward’s Lifesciences LLC, Irvine, CA, USA) and 17–28-Fr venous cannulae (DLP®, Biomedicus®, Medtronic Inc., or RMI®, Edward’s Lifesciences LLC), depending on the patient’s size.

### ECMO management

After the injection of a 50–80 IU/kg heparin bolus, all ECMO patients underwent cannulation using the Seldinger technique in the cardiac catheterization laboratory, which is located next to the ED. When nafamostat mesilate (SK Chemicals Life Science Biz., Seoul, Korea licensed by Torii Pharmaceutical Co., LTD, Tokyo, Japan) was used for anticoagulation during the ECMO, the maintenance dose was held at 0.4–1.5 mg/kg/h to maintain a partial thromboplastin time of 60–80 s [12]. Patients with pulmonary embolism or who had not been placed on continuous renal replacement therapy received heparin. The ECMO flow was held at 3.0–4.0 L/min to maintain a mean blood pressure of >60 mmHg, and we administered norepinephrine or dopamine, as necessary, to maintain the appropriate arterial blood pressure. Among the patients who received E-CPR, those who exhibited a Glasgow Coma Scale score of <9 (eye response: eyes opening to speech, motor: obeys commands, verbal: intubated state) after arriving at the intensive care unit (ICU) were subjected to hypothermic therapy, without the use of sedative drugs, by maintaining their body temperature at 33–34 °C for 24 h [13]. Patients who recovered to a Glasgow Coma Scale of ≥9 were immediately placed on sedatives to increase their body temperature in increments of 0.2 °C/h. During ECMO, the ventilator mode was maintained at a tidal volume of 5 mL/kg, the respiratory rate was maintained at 10/min, and the positive end expiratory pressure was maintained at 4–8 cm H_2_O. The FiO_2_ was held at 0.21–1.0 to maintain 88–100 % arterial oxygen saturation. During VV-ECMO, patients with a clear mental state and a predicted capacity for sputum expectoration were extubated, and oxygen was supplied through a nasal cannula at 3 L/min with awakening ECMO. Patients with acute myocardial infarction (AMI) received percutaneous coronary intervention (PCI) before or after the ECMO. On the day of the PCI, the patients received clopidogrel (300 mg) and aspirin (250 mg), and also received clopidogrel (75 mg) and aspirin (100–200 mg) on the following day. Hematocrit levels and platelet counts of 30–35 % and 50,000–80,000/mL, respectively, were targeted, and a blood transfusion was performed if the level(s) fell below the designated threshold. In cases of heart function recovery without lung function recovery during VA-ECMO, which led to upper body hypoxia (PaO_2_ of <50 mmHg), the ECMO mode was changed to V-AV ECMO if the left ventricle ejection fraction (LVEF) was <30 % (as assessed via two-dimensional echocardiography), or to VV ECMO if the LVEF was >30 %.

The use of VA ECMO was discontinued when echocardiography revealed an LVEF of >30 % at an ECMO flow of 1 L/min. The use of VV ECMO was discontinued if the arterial blood gas analysis indicated a pH of >7.25, a PaO_2_ of 80–120 mmHg, and a PaCO_2_ of 35–45 mmHg at a flow of 1–2 L/min. To patients who were receiving VV ECMO, we delivered a FiO_2_ of 0.21 (supplied via the gas blender) and 0 L/min sweep gas, using ventilator settings of: VT of 6 mL/kg, a respiratory rate of 12/min, and 8 cm of H_2_O positive end expiratory pressure. Alternatively, a FiO_2_ of 0.6 or oxygen (3 L/min) was delivered via a nasal cannula to patients who were awake. Successful ECMO weaning was defined as patient survival of >24 h after ECMO removal, and the primary end-point was defined as patient survival of >28 days.

### Calculation of pre-ECMO SAPS II

SAPS II is a severity score and mortality estimation tool that was developed for patients in medical or surgical ICUs [14, 15]. In this tool, data regarding the worst physiological variables are collected within the first 24 h of ICU admission. In this present study, we modified the SAPS II tool to collect the relevant pre-ECMO data for patients in the ED by collecting data regarding the worst variables within the first 24 h after admission to the ED. In cases of arrest, the heart rate and Glasgow Coma Scale variables were scored at the lowest value (heart rate of <40 beats/min and Glasgow Coma Scale score of <6). If the patient died within 24 h, the urine output variable was estimated by multiplying the hourly urine output (total urine output divided by the total time) by 24.

### Statistical analysis

Statistical analyses were performed using IBM SPSS software (version 21; IBM Corp., Armonk, NY, USA), and differences with a p-value of <0.05 were considered statistically significant. The Mann–Whitney U test was used to evaluate continuous variables, and Pearson’s chi square test or Fisher’s exact test was used for categorical variables. To identify the independent factors that were associated with patient death, we used univariate and multivariate stepwise logistic regression analysis models. The pre-ECMO SAPS II, which was the only significant factor in the multivariate stepwise logistic regression analyses, was subjected to receiver operating characteristic curve (ROC) analysis to identify the optimum cutoff value. Using this cutoff score, we validated the performance of the pre-ECMO SAPS II for predicting 28-day and 60-month mortality, according to the indication, and confirmed the 28-day and 60-month cumulative survival rates using Kaplan-Meier analysis.

## Results

### Baseline and clinical characteristics of the study patients

Among the 65 patients who were included in this presented study, 35 (53.8 %) patients underwent E-CPR, 20 (30.8 %) underwent VA or V-AV ECMO, and 10 (15.4 %) underwent VV ECMO. The 28-day survival rates were 28.6 % in the E-CPR group, 60 % in the VA or V-AV ECMO group, and 90 % in the VV ECMO group (Fig. 1).

The patients’ pre- and during-ECMO characteristics are listed in Tables 1 and 2. The median patient age was 56 years (52 men, 13 women). Regarding the indications for ECMO, AMI (28 patients, 43.1 %) was the most common cause, and was followed by refractory septic shock and traumatic respiratory failure. Among the 51 patients with arrest, the median duration of CPR was 55 min and E-CPR was performed in 35 patients. The overall survival rate in the VV mode was higher than that in the VA or V-AV mode (21/52, 40 % vs. 10/13, 77 %, *p* = 0.018). Compared to the non-survivor group, the survivor group had a longer ECMO duration (72.5 min vs. 143.0 min, *p* = 0.001) and fewer blood transfusions (packed red blood cells: 1.9 units/day vs. 1.0 units/day, *p* = 0.002; fresh frozen plasma: 1.0 units/day vs. 0.3 units/day, *p* = 0.001). However, there were no differences in the use of CCRT, type of anticoagulation treatment, and complications between the survivor and non-survivor groups (Table 3).

**Table 1:** The patient characteristics before extracorporeal membrane oxygenation

Characteristics	Number
Sex, male	52
Age, years	56.0 (42.5, 71.5)
Body mass index (kg/m^2^)	22.9 (21.8, 26.1)
Medical History	
Hypertension	25
Diabetes	22
Coronary artery disease	4
Chronic kidney disease	3
Cerebral vascular accident	3
Pulmonary disease	1
Diagnosis, n (survivors)	
Acute cardiac failure	42 (18)
Acute myocardial infarction	28 (10)
Pulmonary thromboembolism	4 (3)
Unknown cardiac arrest	3 (0)
Other*	7 (5)
Refractory septic shock	9 (3)
Neurogenic circulatory failure	1 (0)
Acute respiratory failure	13 (10)
Traumatic respiratory failure	8 (8)
Sepsis related respiratory failure	3 (1)
Neurogenic pulmonary edema	1 (1)
Status asthmaticus	1 (0)
Arrest, n (survivors)	51 (17)
Out of hospital, n (survivors)	35 (15)
ED, n (survivors)	16 (2)
CPR time, min	55 (20, 72)
CPR-related complications^‡^	22 (34 %)
E-CPR, n (survivors)	35 (10)
Pre-ECMO laboratory findings	
pH	7.09 (6.94, 7.23)
PaO2/FiO2	54.84 (28.9, 80.0)
CK-MB	4.04 (1.80, 12.25)
Troponin-I	0.1 (0.03, 0.86)
BUN	15.4 (11.85, 20.85)
Creatinine	1.20 (0.95, 1.40)
Total bilirubin	0.87 (0.60, 1.38)
AST	55.0 (32.5, 120.5)
ALT	40.0 (24.0, 106.5)
Lactate	8.75 (6.10, 12.73)
IABP	10
SOFA score	13 (11, 14)
Pre-ECMO SAPS II	88 (70, 97)
Door to ECMO time^†^, min	93.0 (56.5, 182.0)
E-CPR	62.0 (49.0, 95.0)
Veno-arterial	157.0 (84.0, 348.0)
Veno-venous	126.0 (102.0, 206.0)

Continuous variables are reported as median (interquartile range)

*CPR* cardiopulmonary resuscitation, *ED* emergency department, *E-CPR* extracorporeal cardiopulmonary resuscitation, *ECMO* extracorporeal membrane oxygenation, *BUN* blood urea nitrogen, *AST* aspartate transaminase, *ALT* alanine transaminase, *IABP* intraaortic balloon pump, *SOFA* Sequential Organ Failure Assessment, *SAPS II* Simplified Acute Physiology Score II

*Hypothermia, malignant arrhythmia, dilated cardiomyopathy, ischemic cardiomyopathy, commotio cordis, abdominal aortic aneurysm rupture

^†^Door to ECMO time: the time from arrival at the ED to ECMO implantation

^‡^Hypoxic brain damage, hemothorax, pulmonary hemorrhage, chest wall compartment syndrome, chylothorax

**Table 2:** The characteristics of survivors and non-survivors during extracorporeal membrane oxygenation support

	Non-survivors	Survivors	*p*-value
	*n* = 34	*n* = 31	
ECMO modes			0.018
VA mode	31	21	
Change from VA to VV	-	1	
Change from VA to V-AV	1	-	
Change from VA to V-AV, and then to VV	-	1	
V-AV mode	2	1	
Change from V-AV to VV	1	-	
VV mode	1	9	
Anticoagulants			0.179
Nafamostat mesilate	30	26	
Heparin	2	5	
ECMO run of ≤24 h	9	1	0.014^a^
Surgery after ECMO	-	4	0.046^a^
ECMO duration, h	72.5 (23.8, 118)	143.0 (93, 212)	0.001
CRRT	18 (60 %)	5 (71 %)	0.687^a^
Daily blood transfusion, unit/d	-	-	
Packed red blood cells	1.9 (0.9, 3.0)	1.0 (0.7, 1.3)	0.002
Fresh frozen plasma	1.0 (0.5, 2.4)	0.3 (0.0, 0.7)	0.001
Platelet concentrate	1.7 (0.0, 5.0)	1.3 (0.0, 3.3)	0.823
Length of stay			
ICU	4 (2, 7)	18 (11, 30)	<0.001
Hospital	4 (2, 7)	28 (20, 51)	<0.001

^a^Fisher’s exact test. Continuous variables are reported as median (interquartile range)

*ECMO* extracorporeal membrane oxygenation, *VA* veno-arterial, *VV* veno-venous, *V-AV* veno-arteriovenous, *CRRT* continuous renal replacement therapy, *ICU* intensive care unit

Surgeries included two pulmonary artery thromboembolectomies, a left upper lobectomy, and an aneurysm clipping via a left frontotemporal craniectomy

**Table 3:** A comparison of complications among survivors and non-survivors

	Non-survivors	Survivors	p-value
	n = 34	n = 31	
Cannula-related complications	2	2	1.000^a^
Cannula site bleeding	1	-	
Leg ischemia	1	1	
Thrombosis	-	1	
Acute renal failure	12	9	0.590
SIRS	1	-	1.000^a^
Gastrointestinal bleeding	3	2	1.000^a^
Cholecystitis	-	1	0.477^a^
Intracranial hemorrhage	2	-	1.000^a^

^a^Fisher’s exact test. SIRS, systemic inflammatory response syndrome

### Predictors for mortality in patients with ECMO support at the ED

In the logistic regression analyses, all 28-day variables that were under consideration (hypertension, cardiac arrest on arrival, E-CPR, CPR time, ECMO VA mode, lactate, SOFA score, pre-ECMO SAPS II, and CPR-related complications) were significantly higher in the non-survivors, compared to the values in the survivors. However, in the multivariate analysis, only pre-ECMO SAPS II was a statistically significant predictor (odds ratio [OR]: 1.189; 95 % confidence interval [CI]: 1.032–1.370; *p* = 0.016) (Table 4).

**Table 4:** Univariate and multiple logistic regression analysis of pre-extracorporeal membrane oxygenation predictors of 28-day mortality

Variable	Univariate analysis			Multivariate analysis		
	OR	95 % CI	*p*-value	OR	95 % CI	*p*-value
Hypertension	3.857	1.312-11.337	0.014	1.921	0.123-29.942	0.641
Cardiac arrest on arrival	3.392	1.220-9.431	0.019	0.012	0.000-1.896	0.087
E-CPR	5.833	1.998-17.028	0.001	1.078	0.055-21.099	0.960
CPR time	1.021	1.000-1.041	0.047	1.002	0.955-1.051	0.941
VA/VV ECMO	14.211	1.665-121.316	0.015	4.626	0.023-925.946	0.571
Lactate	1.334	1.109-1.605	0.002	1.345	0.885-2.045	0.165
SOFA score	1.430	1.146-1.785	0.002	1.191	0.539-2.633	0.665
CPR related Cx	7.594	2.181-26.437	0.001	1.138	0.082-15.840	0.923
Pre-ECMO SAPS II	1.131	1.059-1.207	<0.001	1.189	1.032-1.370	0.016

In order to solve the multicollinearity problem, we excluded the variables that are included in SAPS II from the logistic regression analysi

*E-CPR* extracorporeal cardiopulmonary resuscitation, *CPR* cardiopulmonary resuscitation, *VA* veno-arterial, *VV* veno-venous, *ECMO* extracorporeal membrane oxygenation, *SOFA* Sequential Organ Failure Assessment, *SAPS II* Simplified Acute Physiology Score II, *Cx* complications

**Fig. 2:**
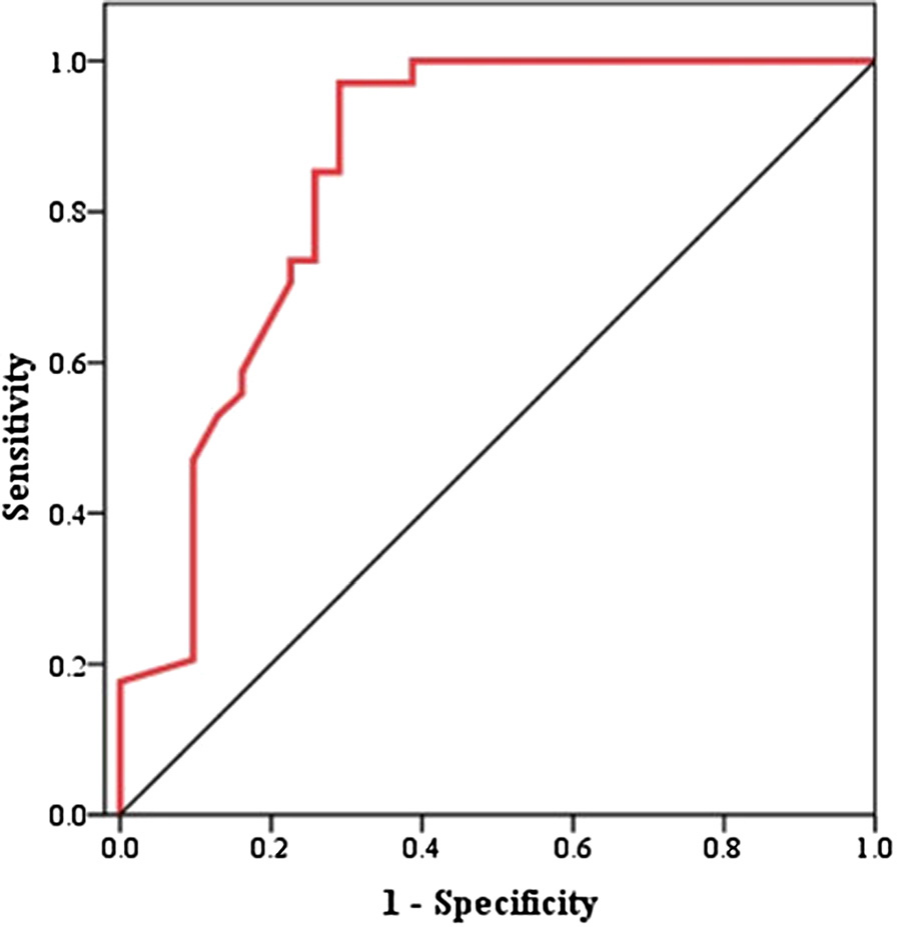
Receiver operating characteristics curves for pre-ECMO SAPS II in predicting 28-day mortality. The figure shows the receiver operating characteristics curve for pre-extracorporeal membrane oxygenation (ECMO) Simplified Acute Physiology Score (SAPS) II scoring, with an area under the curve of 0.852 (*p* < 0.001; 95 % CI: 0.753–0.951). At a SAPS II cutoff value of 80, the sensitivity and 1-specificity were 0.971 and 0.290, respectively

The clinical relevance of pre-ECMO SAPS II as a predictor for 28-day mortality was further confirmed by the subsequent ROC analyses. The area under the ROC curve for pre-ECMO SAPS II was 0.852 (95 % CI: 0.753–0.951, *p* < 0.001), and the optimal cutoff value for pre-ECMO SAPS II was 80 (sensitivity of 97.1 % and specificity of 71.0 %), respectively (Fig. 2).

### Validating the performance of pre-ECMO SAPS II for 28-day and 60-month mortality

**Fig. 3:**
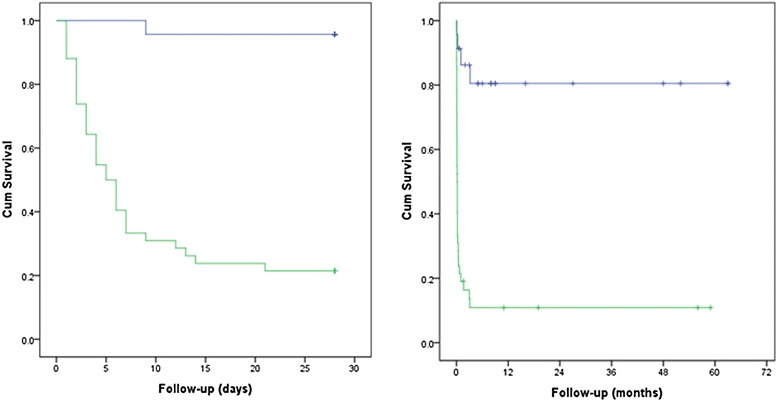
Kaplan-Meier analysis for a pre-ECMO SAPS II score of 80 in predicting 28-day and 60-month mortality. The risk of 28-day and 60-month mortality among patients with SAPS II of ≤80 was lower than that of patients with SAPS II of >80 (both, *p* < 0.001)

The cutoff value of 80 exhibited a statistically significant difference for the 28-day and 60-month mortality rates in the overall, E-CPR, and VA groups (28-day: *p* < 0.001, *p* = 0.004, *p* = 0.005; 60-month: *p* < 0.001, *p* = 0.004, *p* = 0.020) (Table 5). In the Kaplan-Meier analysis, the 28-day and 60-month cumulative survival rates were lower among the patients with a pre-ECMO SAPS II of ≤80, compared to those with a pre-ECMO SAPS II of >80 (both, *p* < 0.001) (Fig. 3).

**Table 5:** Validation of SAPS II for 28-day and 60-month mortality according to the indication

	Overall (*N* = 65)			E-CPR (*N* = 35)			VA and V-AV (*N* = 20)			VV (*N* = 10)		
	Survivor (*N* = 31)	Death (*N* = 34)	*p-*value <0.001	Survivor (*N* = 10)	Death (*N* = 25)	*p-*value 0.004	Survivor (*N* = 11)	Death (*N* = 8)	*p-*value 0.005	Survivor (*N* = 9)	Death (*N* = 1)	*p-*value 0.100
28-day mortality												
SAPS II ≤80	22 (71.0)	1 (2.9)		5 (50.0)	1 (4.0)		8 (66.7)	0 (0.0)		9 (100.0)	0 (0.0)	
SAPS II >80	9 (29.0)	33 (97.1)		5 (50.0)	24 (96.0)		4 (33.3)	8 (100.0)		0 (0.0)	1 (100.0)	
60-month mortality												
SAPS II ≤80	19 (79.2)	4 (9.8)		4 (66.7)	2 (6.9)		7 (70.0)	1 (10.0)		8 (100.0)	1 (50.0)	
SAPS II >80	5 (20.8)	37 (90.2)		2 (33.3)	27 (93.1)		3 (30.0)	9 (90.0)		0 (0.0)	1 (50.0)

## Discussion

The present study’s results demonstrated that pre-ECMO SAPS II is an independent predictor for 28-day mortality among patients with ECMO support at the ED. In the validation of its performance, the cutoff value of 80 exhibited statistically significant differences for the 28-day and 60-month mortality rates, especially in the E-CPR and VA ECMO groups. Therefore, the pre-ECMO SAPS II could be helpful for selecting patients with refractory acute circulatory and/or respiratory failure who will respond to ECMO support at the ED.

The selection of appropriate candidates for ECMO support requires serious consideration, due to the high economic cost and labor intensive nature. A small number of recent studies have attempted to develop a scoring system for identifying these patients. Schmidt et al. evaluated the PRESERVE and RESP scores in their survival prediction model for patients who received ECMO support in the ICU [10, 11]. However, there are several limitations to applying these systems for patients in the ED. First, those studies only enrolled patients with respiratory failure, which inevitably led to most of the ECMO being VV mode (PRESERVE VA mode: 133/140 [95 %] and RESP VA mode: 1,928/2,355 [82 %]). However, similar to our results, VA modes were applied more than VV modes for patients who received ECMO support in the ED. Second, during E-CPR and VA ECMO, it is difficult to assess mechanical ventilation duration and settings. Third, Klinzing et al. demonstrated that the RESERVE and RESP scoring systems failed to predict mortality for patients who were receiving VA ECMO [16]. In the present study, pre-ECMO SAPS II significantly predicted the survival of patients who received VA ECMO and E-CPR. The VV ECMO group exhibited a similar tendency, although the result was not statistically significant.

Our survival rate results demonstrated better outcomes, compared to those of previous studies. We suggest that the discrepancies between these findings may be related to patient enrollment. The majority of indications for VA or V-AV ECMO were for acute cardiac failure, especially AMI, and only a relatively small number of patients with septic shock (which has a poor prognosis) were included. In contrast, the major indication for VV ECMO was traumatic acute respiratory distress syndrome, which has a favorable prognosis.

Given the ongoing progress in the development of ECMO equipment, cannula can easily and safely be implanted into patients’ peripheral vessels, which facilitates the rapid application of ECMO. This rapid emergent cardiopulmonary assistance technique can also facilitate the appropriate testing and diagnosis, and thereby allow for appropriate treatment [17–20]. In the present study, 43 % of the patients who received ECMO support at the ED had presented with complications of refractory cardiogenic shock or cardiac arrest due to AMI. These patients underwent PCI with ECMO, which provided a 35.7 % patient survival rate. Therefore, AMI that is accompanied by cardiogenic shock, or cases of cardiac arrest that present with a high risk of mortality when using conventional treatment methods, may be suitable for ECMO, which can facilitate appropriate diagnosis and treatment, and improve patient survival rates [6, 21–25]. Furthermore, other studies have reported that, in cases of in- and out-of-hospital cardiac arrest with cardiac origin, E-CPR provided a survival rate of 32.1–34.1 %, which is higher than that obtained using conventional CPR [5, 7, 26].

Among the four patients with acute massive pulmonary embolism who received ECMO in our study, one patient presented with out-of-hospital cardiac arrest and was diagnosed with a massive pulmonary embolism after the E-CPR. Unfortunately, this patient subsequently died of hypoxic brain damage. Two of the three survivors underwent pulmonary embolectomy after the ECMO, while the other surviving patient underwent successful medical treatment. For patients in near-fatal condition (due to massive pulmonary embolisms), the survival rate of medical or surgical treatment after ECMO is 62–70 % [27, 28]. In these cases, the clinical decision to administer ECMO, the reversibility of the underlying disease, and the patient’s condition after ECMO all affect the prognosis, and an aggressive diagnosis and treatment of the underlying disease are critical to patient survival [22].

In this present study, 33 % of the patients with acute sepsis-related circulatory and/or respiratory failure ultimately survived. Although the use of ECMO in patients with sepsis remains controversial, it may be effective for use in a select group of patients [8, 29]. In addition, the survival rate for patients with acute respiratory failure was 77 % (10/13), compared to 40 % (21/52) for patients with acute circulatory failure, which is due to the fact that all 8 patients with traumatic respiratory failure who received ECMO ultimately survived. Therefore, VV ECMO is very effective in treating patients with severe thoracic trauma and acute lung failure from various causes [3, 9].

As patients in the ED require rapid diagnosis and treatment, it is difficult to determine appropriate ECMO implantation standards and identify patients with acute cardiopulmonary failure who will not respond to conventional treatment. Although pre-ECMO SAPS II is widely used to predict mortality among ICU patients, this can be also useful for predicting ED patient mortality in cases of cardiovascular and respiratory illnesses [30]. Although the median pre-ECMO SAPS II in our study was 88, which predicted an extremely high mortality rate (96.1 %), the 28-day and 60-month overall mortality rates for our patients were 52.3 % and 63.1 %. Furthermore, these mortality rates for patients with a pre-ECMO SAPS II of ≤80 were 2.9 % and 20.8 %, respectively. Therefore, we believe that the pre-ECMO SAPS II score can lead to the appropriate selection of patients for ECMO support, and improve patient outcomes.

This study had several limitations. First, it was based at a single center, which limits the generalizability of the findings. Second, the study population was small, and the underlying diseases were diverse. Third, in VA ECMO, a relatively small number of patients with refractory septic shock were enrolled in this study. Thus, further studies are needed to determine whether our findings can be accurately applied to these patients. Finally, we performed a retrospective analysis, and additional prospective multicenter studies are needed to validate our findings.

## Conclusions

Pre-ECMO SAPS II could be helpful for selecting patients with refractory acute circulatory and/or respiratory failure who will respond to ECMO support at the ED.
